# Supplementary material for: Heat-Mode Excitation in a Proximity Superconductor
Source: Nanomaterials (Basel). 2022 Apr 25;12(9):1461. doi: 10.3390/nano12091461 (PMC9101060; doi:10.3390/nano12091461)
Supplement: Supplementary file 1 [file nanomaterials-12-01461-s001.zip › nanomaterials-1670706-supplementary.pdf]

Heat-mode excitation in a proximity superconductor
Supplemental Materials

A.O. Denisov,[1,2] A.V. Bubis,[3,1] S.U. Piatrusha,[1] N.A. Titova,[4] A.G. Nasibulin,[3]
J. Becker,[5] J. Treu,[5] D. Ruhstorfer,[5] G. Koblmüller,[5] E.S. Tikhonov,[1] and V.S. Khrapai[1,6]

[1] *Osipyan Institute of Solid State Physics, Russian Academy of Sciences, 142432 Chernogolovka, Russia*
[2] *Department of Physics, Princeton University, Princeton, New Jersey 08544, USA*
[3] *Skolkovo Institute of Science and Technology, Nobel street 3, 121205 Moscow, Russian Federation*
[4] *Moscow Pedagogical State University, 29 Malaya Pirogovskaya St, 119435 Moscow, Russia*
[5] *Walter Schottky Institut, Physik Department, and Center for Nanotechnology and Nanomaterials, Technische Universität München, Am Coulombwall 4, Garching 85748, Germany*
[6] *National Research University Higher School of Economics, 20 Myasnitskaya Street, 101000 Moscow, Russia*

This file contains supplementary information for the main text, including the following figures:

Figure S1: Sketch of the experimental setup
Figure S2: Calibration via equilibrium noise
Figure S3: Shot-noise analysis
Figure S4: Additional data in device NSN-I: local conductance
Figure S5: Additional data in device NSN-II: local conductance
Figure S6: Additional data in device NSN-I: non-local conductance
Figure S7: Additional data in device NSN-II: non-local conductance
Figure S8:. Effective resistance model for NW/S interface
Figure S9: T-dependence in the linear response regime and calibration of the resistive thermometry
Figure S10: T-dependence beyond the linear response regime
Figure S11: Analytical model: layout and EED
Figure S12: Analytical model: results
Figure S13: Comparison of the non-local noise thermometry and resistive thermometry
Figure S14: Superconducting critical temperature of the Al-film

Noise and charge transport measurements

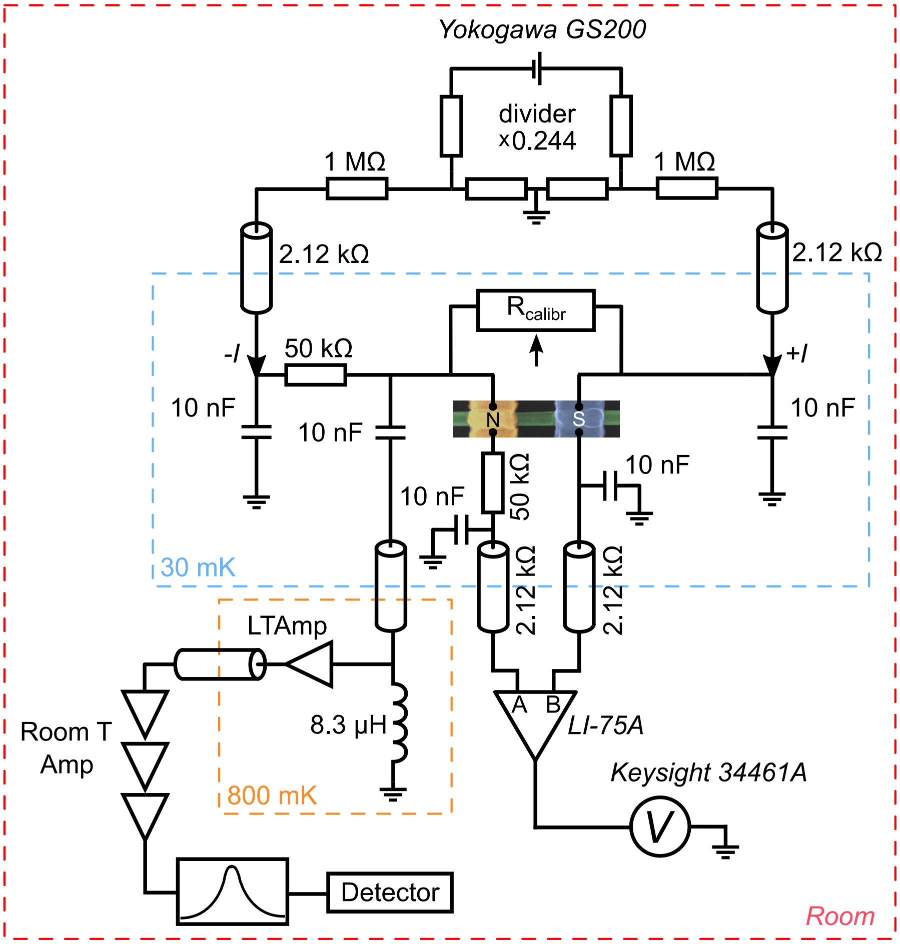

FIG. S1. **Sketch of the experimental setup.**

DC and low frequency AC transport measurements are carried out using symmetric current bias scheme with divider shown in Fig. S1. We use quasi-4-terminal setup thus excluding wiring and filtering contribution into measured voltage signal. We use SR-7265 lock-in for resistive thermometry with typical modulation current of 2 nA, f = 19.3 Hz, time constant = 2s, AC gain 30 dB and filter slope of 24 dB/oct.
The noise spectral density was measured using the home-made low-temperature amplifier (LTamp) with a voltage gain of about 10 dB and the input current noise of $\sim 2\text{–}6\times10^{-27}$ A^2/Hz. The voltage fluctuations on a 25 kΩ load resistance were measured near the central frequency 14.2 MHz (±0.6 MHz for −3 dB point) of a resonant circuit at the input of the LTAmp. The output of the LTamp was fed into the low noise 75 dB gain room temperature amplification stage followed by a hand-made analogue band-pass filter and a power detector. The setup was calibrated using HEMT ATF-35143 as adjustable load $R_{\text{calib}} = 50\ \Omega\ \rightarrow\ > 100$ MΩ for the equilibrium Johnson-Nyquist noise thermometry. Except for the periods of calibration, the transistor was always kept pinched off. Unless otherwise stated, the measurements were performed in a cryogenic free Bluefors dilution refrigerator BF-LD250 at a bath temperature of 30 mK.

Johnson-Nyquist noise thermometry

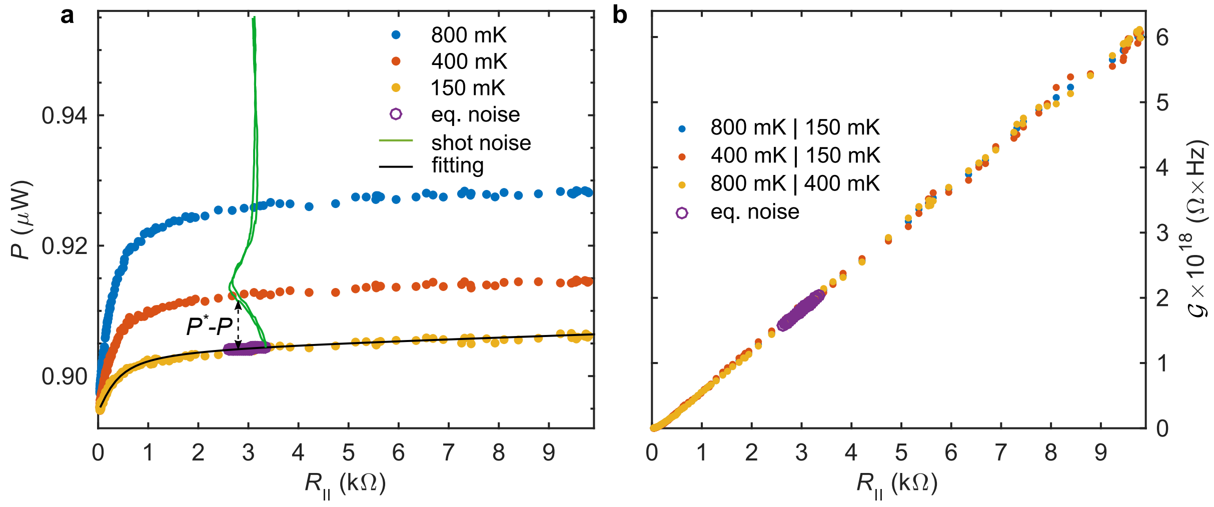

FIG. S2. **Calibration via equilibrium noise.** (a) Power released on the detector as a function of total load resistance $R_{||}$ measured at different bath temperatures (see electron temperature in the legend). Green solid line shows typical reflected shot-noise signal at applying bias current through the device (NSN - I, $V_g = 40$ V). Black solid line is fit using Nyquist relation. Purple circles shows bias-dependent gain and equilibrium noise. (b) Total gain of the setup. Three sets of points correspond to three different combinations of $T_1|T_2$ (see text below).

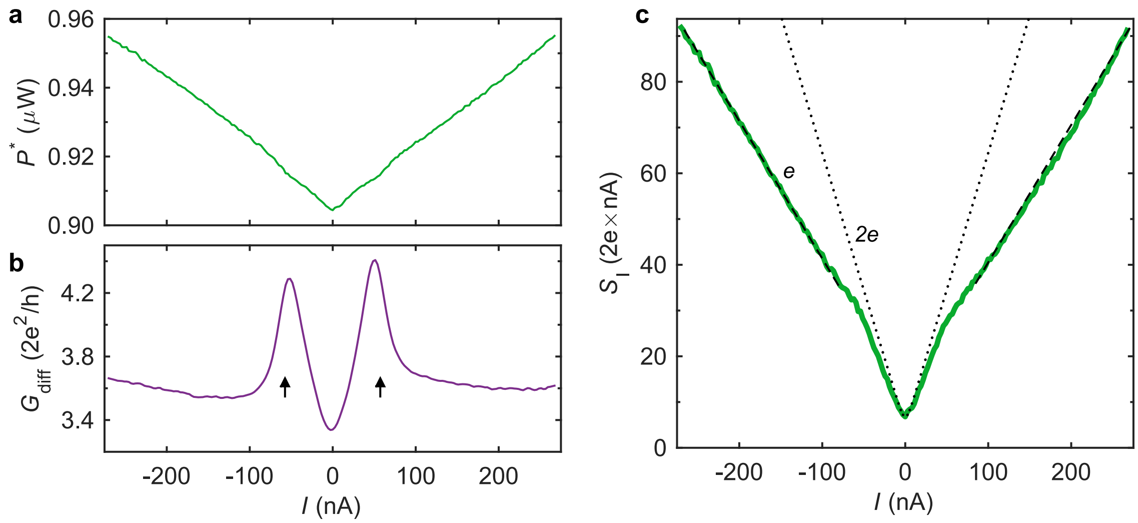

FIG. S3. **Shot-noise analysis.** (a) Power dissipated in the detector is plotted as a function of current through the sample. (b) Differential conductance of the NS junction. Arrows mark superconducting gap $\pm\Delta$. (c) Extracted current noise spectral density. Dashed line corresponds to Fano-factor $F = S_I/2eI = 0.30$ and $e^* = e$, dotted line is for doubled charge - $F = 0.30$ and $e^* = 2e$.

We calibrate our setup using equilibrium noise thermometry. At zero current through the device, we are able to change a value of $R_{||} = (G_{\text{diff}} + R^{-1}_{25\text{k}\Omega} + R^{-1}_{\text{calibr}})^{-1}$ drastically, where G_{diff} is a differential resistance (zero bias) of the sample shown in Fig. S3b. Power released on the detector after all amplification stages :

$$P(R_{||}) = \Big(\frac{4k_\text{B}T}{R_{||}} + S_\text{I}^{\text{Amp}}\Big) \int \frac{G \times Tr^{\text{filter}}(f)}{R_{||}^{-2} + |Z_{\text{LC}}|^{-2}} df + P_0 = \mathcal{G}(R_{||})\Big(\frac{4k_\text{B}T}{R_{||}} + S_\text{I}^{\text{Amp}}\Big) + P_0 \tag{S1}$$

where G is an unknown total gain, Z_{LC} - complex impedance of the LC contour, $Tr^{\text{filter}}(f)$ - transmission characteristic of the band-pass filter, S_I^{Amp} and P_0 - parasitic current noise and background of the low-temperature amplifier. After an integration over frequency, we can use generalized value for gain $\mathcal{G}(R_{||})$ which can be extracted by measuring $P(R_{||})$ at different bath temperatures:

$$\mathcal{G}(R_{||}) = \frac{P(R_{||}, T_1) - P(R_{||}, T_2)}{T_1 - T_2} \frac{R_{||}}{4k_\text{B}} \tag{S2}$$

When we apply current through the sample, the crossover from thermal to non-equilibrium shot noise (see solid green curve in Fig. S2a and Fig. S3a) appears. Depending on the bias current, $R_{||}(I)$ is changing thus making $\mathcal{G}(R_{||})$ bias-dependent (see purple symbols in Fig. S2a, b). Desired current noise of the sample S_I contributes to the total power as follows (transistor is pinched off $R_{\text{calib}} > 100$ MΩ) :

$$P^*(R_{||}) = \mathcal{G}(R_{||})\Big(\frac{4k_\text{B}T}{R_{25\text{k}\Omega}} + S_\text{I} + S_\text{I}^{\text{Amp}}\Big) + P_0, \quad S_\text{I} = \frac{P^*(R_{||}) - P(R_{||})}{\mathcal{G}(R_{||})} + 4k_\text{B}TG_{\text{diff}} \tag{S3}$$

Finalized current spectral density curve is shown in Fig. S3c.

Local charge transport

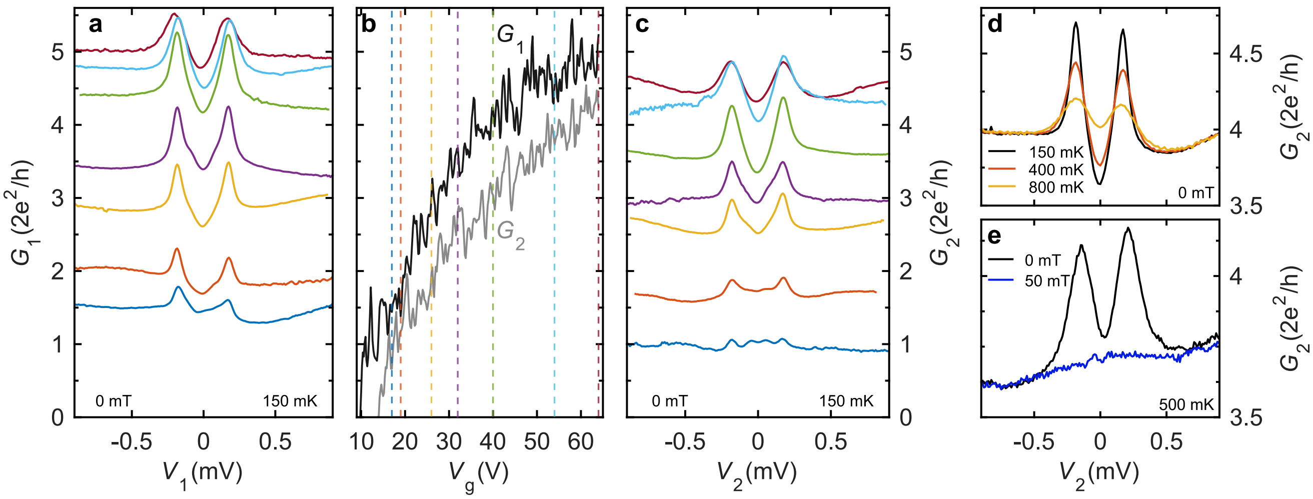

FIG. S4. **Additional data in device NSN-I: local conductance.** (a) and (c) Local spectral conductance of the left and right NS junctions correspondingly, measured at different back-gate voltages $V_{\mathrm{g}} =$ 17, 19 ,26, 32, 40, 54, 64 V from bottom to top. (b) Linear-response conductance is plotted as function of V_{g}. Dashed lines of corresponding colors point certain values of back-gate voltages from (a) and (c). (d) and (e) Temperature and magnetic field dependence of the spectral conductance measured at constant $V_{\mathrm{g}} = 41$ and 50 V correspondingly.

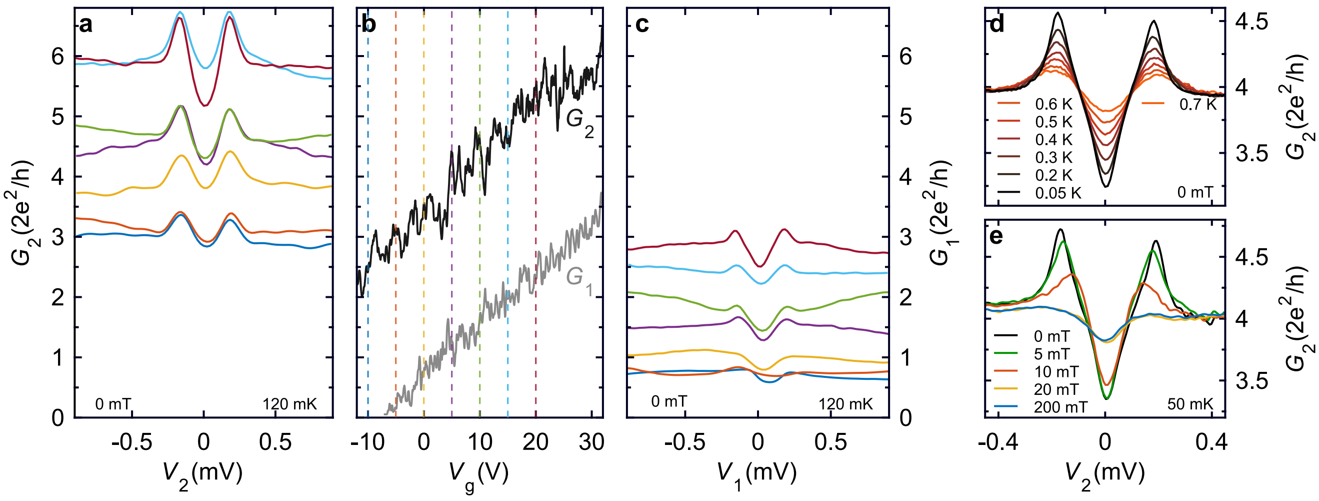

FIG. S5. **Additional data in device NSN-II: local conductance.** (a) and (c) Local spectral conductance of the left and right NS junctions correspondingly, measured at different back-gate voltages $V_{\mathrm{g}} = \ -10,\ -5\ ,0,\ 5,\ 10,\ 15,\ 20$ V from bottom to top. (b) Linear-response conductance is plotted as function of V_{g}. Dashed lines of corresponding colors point certain values of back-gate voltages from (a) and (c). (d) and (e) Temperature and magnetic field dependence of the spectral conductance measured at constant $V_{\mathrm{g}} = 0$ V.

Non-local charge transport

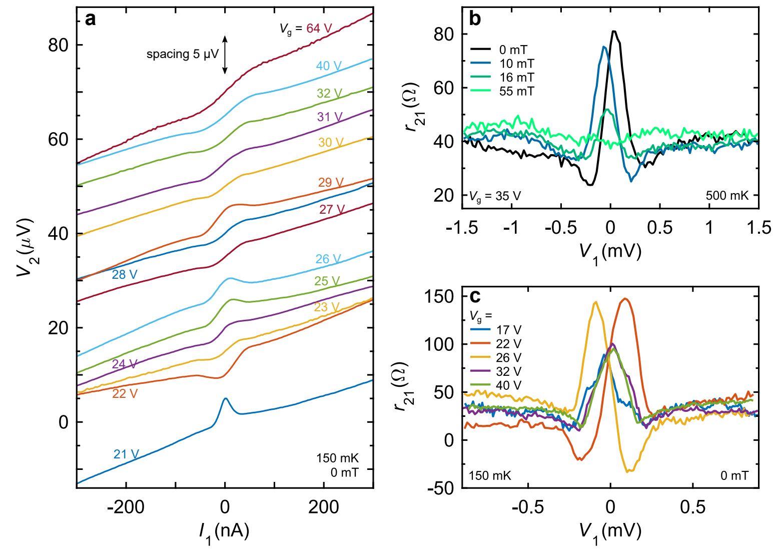

FIG. S6. **Additional data in device NSN-I: non-local conductance.** (a) Non-local I/V characteristics measured at different back-gate voltages. For convenience, curves are shifted vertically with spacing of 5 μV. (b) and (c) Differential non-local resistance $r_{21} = dV_2/dI_1,\ I_2 = 0$ at different magnetic fields and back-gate voltages correspondingly.

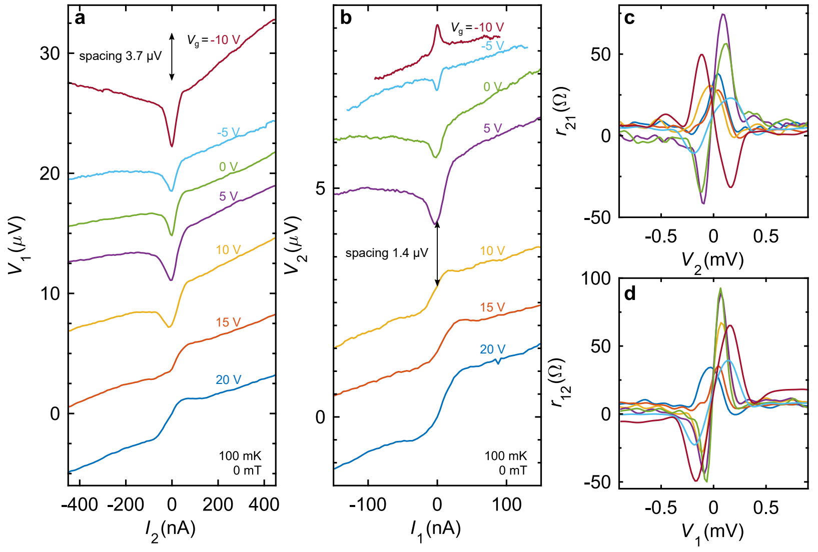

FIG. S7. **Additional data in device NSN-II: non-local conductance.** (a) and (b) Non-local I/V characteristics of both NS junctions measured at different back-gate voltages. For convenience, curves are shifted vertically with spacing of 3.7 μV and 1.4 μV correspondingly. (c) and (d) Differential non-local resistance $r_{21} = dV_2/dI_1,\ I_2 = 0$ and $r_{12} = dV_1/dI_2,\ I_1 = 0$ at different back-gate voltages. Colors match those from (a) and (b).

Current transfer length estimation

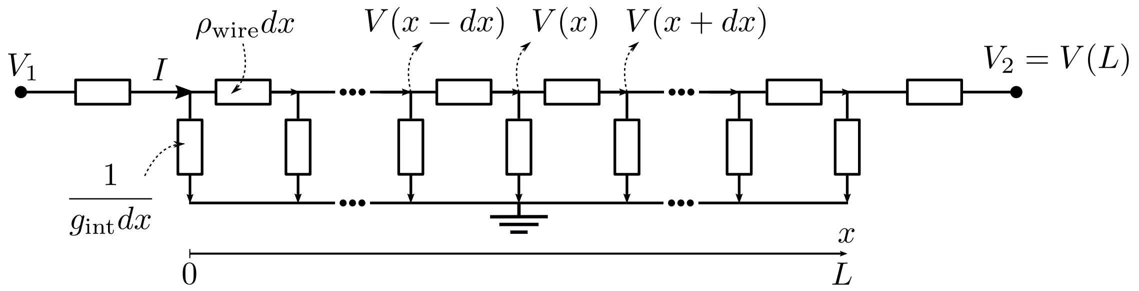

FIG. S8. **Effective resistance model for nanowire/superconductor interface.**

To estimate the characteristic length of charge overflow within grounded S terminal $l_{\rm T}$ we use circuit shown in fig. S8. Here $\rho_{\rm wire}$ and $g_{\rm int}$ are resistance of the nanowire (NW) and conductivity of interface per unit length respectively. In the continuous limit we can write current conservation for each point along NW/S interface:

$$\frac{V(x+dx)-V(x)}{\rho_{\rm wire}dx}+\frac{V(x-dx)-V(x)}{\rho_{\rm wire}dx}=\frac{V(x)dx}{1/g_{\rm int}}$$

$$\frac{d^2V(x)}{dx^2}=\frac{V(x)}{l_{\rm T}^2},\quad l_{\rm T}=\frac{1}{\sqrt{\rho_{\rm wire}g_{\rm int}}}$$

Boundary conditions including one that normal terminal N2 is floating and no current flow into it.

$$\left.\frac{dV(x)}{dx}\right|_{x=0}=-\rho_{\rm wire}I,\quad \left.\frac{dV(x)}{dx}\right|_{x=L}=0$$

Solving elementary Neumann problem we can find non-local rsistance r_{21}:

$$r_{21}=\frac{V(L)}{I}=\frac{l_{\rm T}\rho_{\rm wire}}{\sinh(\frac{L}{l_{\rm T}})}$$

For two measured devices (NSN-I, NSN-II) we have $r_{21}\approx 40,\ 10\,\Omega$ and $L\approx 200,\ 300\,\text{nm}$ respectively, thus $l_{\rm T}\approx 75\,\text{nm}$.

Temperature dependence of differential conductance.

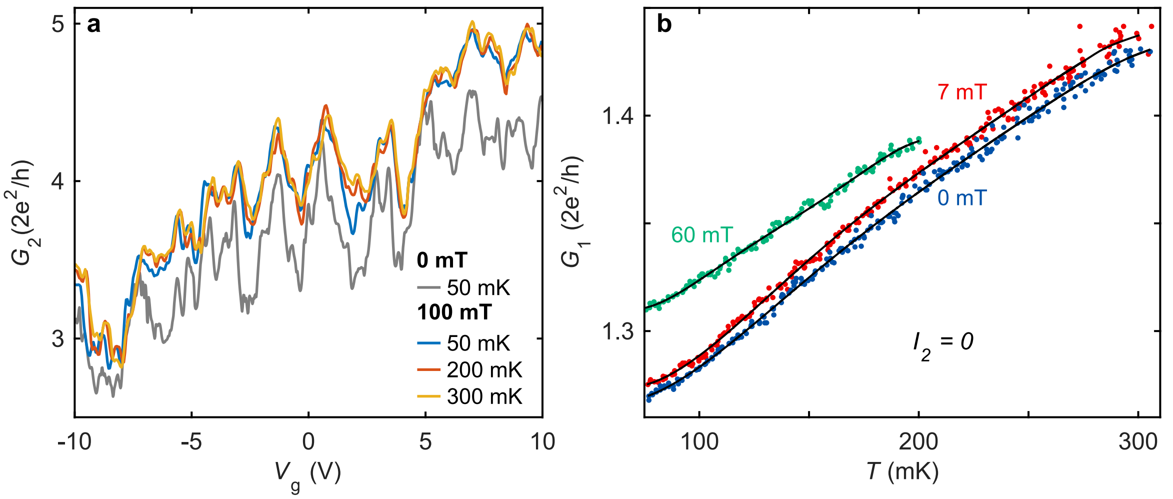

FIG. S9. **T-dependence in the linear response regime and calibration of the resistive thermometry.** (a) Linear-response conductance of a single NS junction (NSN - II device) measured at different bath temperatures. Overall increasing dependence with reproducible UCF persists up to the shift $\sim 10\%$ when large magnetic field ($100\,\mathrm{mT} > B_c$) is applied. Such zero-bias deep is more evident from differential conductance data measured at $V_g = 0\,\mathrm{V}$ and $I_2 = 0$ shown in (b). Solid lines are smooth polynomial fits we use for the resistive thermometry.

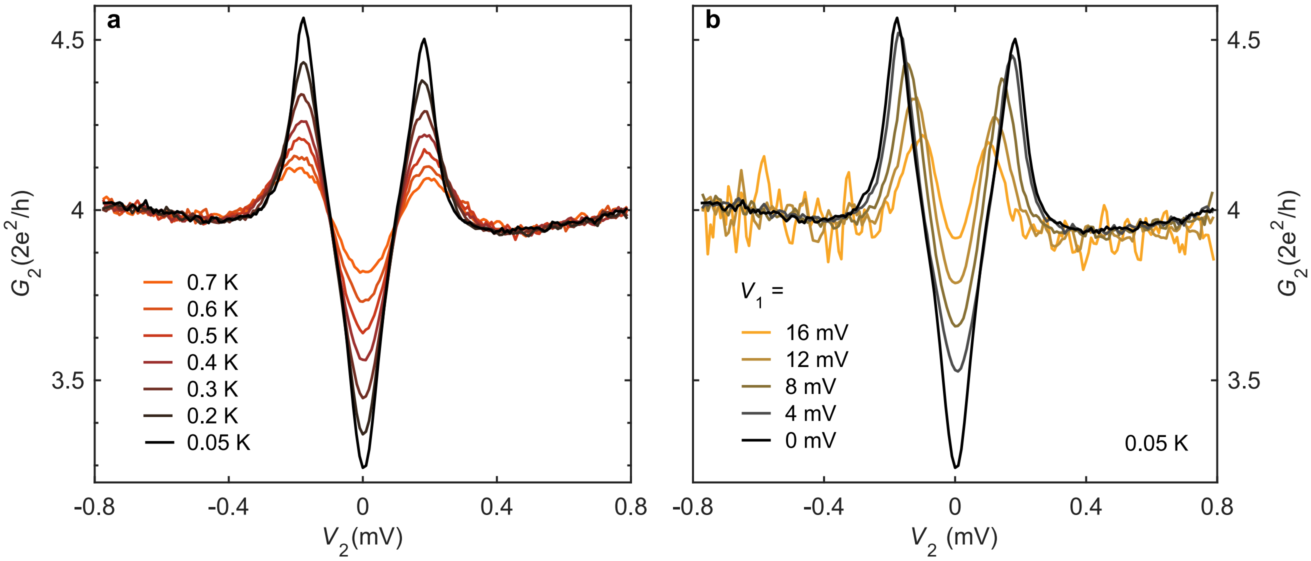

FIG. S10. **T-dependence beyond the linear response regime.** (a) Spectral conductance measured at constant $V_g =$ 0 V (NSN - II device) and different bath temperatures. (b) Spectral conductance measured at constant $V_g =$ 0 V, bath temperature but different "heating" voltages across the adjacent NS junction.

Analytical model

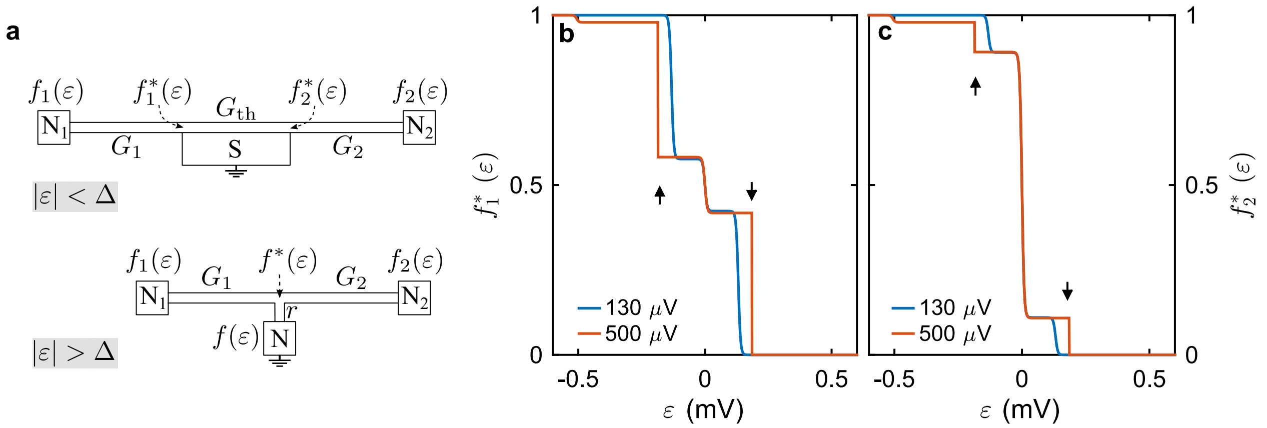

FIG. S11. **Analytical model: layout and EED.** (a) Schematics of the analytical model, separately for sub-gap and above-gap quasiparticle energies. (b) - (c) Calculated EEDs near two ends of the proximitized sections for $V_1 = 130\ \mu$V and $500\ \mu$V ($T = 50$ mK, $G_{\rm th} =\ 1.17 \times 2e^2/h,\ r = 64\ \Omega$). Arrows mark the superconducting gap of Al, $\Delta = 180\ \mu V$.

The sketch of analytical model we use to fit experimental data is shown in fig.3a. Since we operate in the non-linear $\varepsilon_T \sim k_{\rm B}T \ll eV$ regime, where $\xi_T = \hbar D/L^2 \approx 15\,\mu$V is the Thouless energy, we can neglect the penetration of the condensate into NW and consider it just as a normal metal with a non-equilibrium electronic energy distribution (EED). Distribution functions near the N terminals $f_{1/2}(\varepsilon)$ are equilibrium Fermi-Dirac functions with local temperature and chemical potential of the corresponding terminal. EED near S should satisfy Andreev conditions for the energies below the gap:

$$f^*_{1/2}(\varepsilon) = \begin{cases} 1 - f^*_{1/2}(-\varepsilon) & |\varepsilon| < \Delta \\ (\exp(\varepsilon/k_{\rm B}T) + 1)^{-1} & |\varepsilon| > \Delta \end{cases} \tag{S4}$$

Following Nagaev and Buttiker [S1] we separately find electron distribution functions for energies below and above the gap and then sew them together. With EEDs near S terminal in hand, we are able to calculate current noise spectral density in both NS junction [S2] i.e. reflected (R) and transmitted (T) shot noise:

$$S_{\rm R/T} = \frac{2}{3}G_{1/2}\int \Big[2f_{1/2}(\varepsilon)(1-f_{1/2}(\varepsilon)) + 2f^*_{1/2}(\varepsilon)(1-f^*_{1/2}(\varepsilon)) + f_{1/2}(\varepsilon)(1-f^*_{1/2}(\varepsilon)) + f^*_{1/2}(\varepsilon)(1-f_{1/2}(\varepsilon))\Big]d\varepsilon \tag{S5}$$

Sub-gap ($|\varepsilon| < \Delta$)

First we calculate for energies within the superconducting gap. Since noise temperature gradient is zero at N/S interface, then the correction to the noise temperature due to the finite r is of the second order and we can neglect it. In order to find the energy distributions $f_1^*(\varepsilon)$ and $f_1^*(\varepsilon)$ at the two ends of the proximitized wire section, one has to fulfill the continuity of the heat fluxes in these points at any given ε. In this way we get the following two equations:

$$G_2[f_2(\varepsilon) - f_2^*(\varepsilon)] - G_2[f_2(-\varepsilon) - f_2^*(-\varepsilon)] = G_{\rm th}[f_2(\varepsilon) - f_1^*(\varepsilon)] - G_{\rm th}[f_2(-\varepsilon) - f_1^*(-\varepsilon)] \tag{S6}$$

$$G_1[f_1(\varepsilon) - f_1^*(\varepsilon)] - G_1[f_1(-\varepsilon) - f_1^*(-\varepsilon)] = G_{\rm th}[f_2(\varepsilon) - f_1^*(\varepsilon)] - G_{\rm th}[f_2(-\varepsilon) - f_1^*(-\varepsilon)] \tag{S7}$$

where the terms with ε correspond to the particle heat flux and the terms with $-\varepsilon$ to the hole heat flux. The equations (S6) and (S7) are for the right and the left NS interface respectively. For convinience lets introduce $F(\varepsilon) \equiv f(\varepsilon) - f(-\varepsilon)$, then:

$$F_1^*(\varepsilon) = \frac{G_1G_2F_1(\varepsilon) + G_{\rm th}G_1F_1(\varepsilon) + G_{\rm th}G_2F_2(\varepsilon)}{G_{\rm th}G_1 + G_{\rm th}G_2 + G_1G_2},\ F_2^*(\varepsilon) = \frac{G_1G_2F_2(\varepsilon) + G_{\rm th}G_2F_2(\varepsilon) + G_{\rm th}G_1F_1(\varepsilon)}{G_{\rm th}G_1 + G_{\rm th}G_2 + G_1G_2} \tag{S8}$$

Since $f_i^*(\varepsilon) = 1 - f_i^*(-\varepsilon)$, we can easily find $f_i^* = (1 + F_i^*)/2$

Above-gap ($|\varepsilon| < \Delta$)

To calculate for energies above the superconducting gap, we need to take into account the fact that now there is a finite gradient of noise temperature near N/S interface. Then correction to $T_{\rm N}$ due to the finite r is of the first order and we can not neglect it. Fortunately, for energies above the gap, S terminal acts as regular normal lead, so we can assume $f^*(\varepsilon) = f^*_{1/2}(\varepsilon)$. In order to find $f^*(\varepsilon)$, one has to fulfill current conservation law for each energy:

$$G_2[f_2(\varepsilon) - f^*(\varepsilon)] + G_1[f_1(\varepsilon) - f^*(\varepsilon)] = r^{-1}[f^*(\varepsilon) - f_0(\varepsilon)] \tag{S9}$$

where $f_0(\varepsilon)$ is a Fermi-Dirac distribution in in the normal state of grounded Al terminal and r is the interface resistance.

$$f^*(\varepsilon) = \frac{G_1 f_1(\varepsilon) + G_2 f_2(\varepsilon) + 1/r f_0(\varepsilon)}{G_1 + G_2 + r^{-1}} \tag{S10}$$

Reflected and Transmitted shot noise

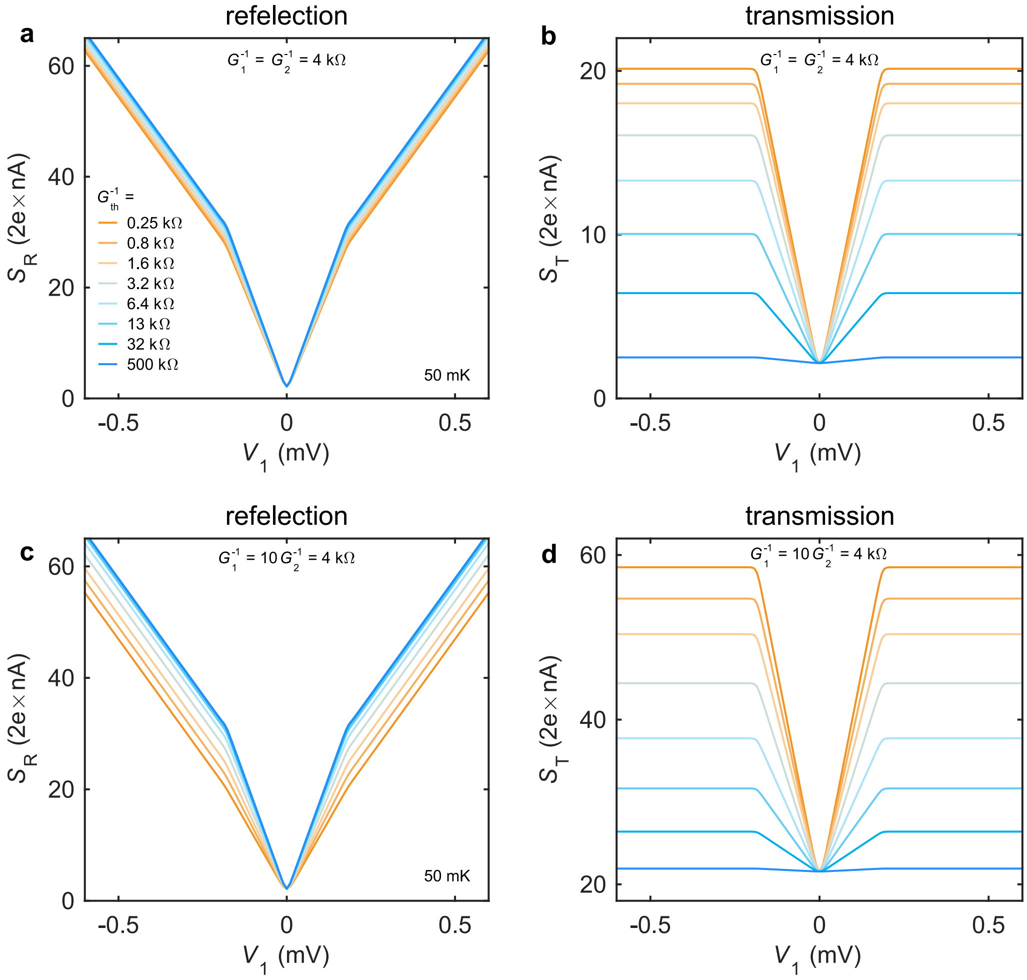

FIG. S12. **Analytical model: results.** (a) - (b) Calculated shot noise in reflection and transmission configurations for $G_1 = G_2$ and various $G_{\rm th}$. (c) - (d) Calculated shot noise in reflection and transmission configurations for $G_2 = 10G_1$ and various $G_{\rm th}$.

Calculated with (S5) reflected and transmitted shot noise are plotted in fig. S12. For clarity, we consider a case of the perfect interface $r = 0$ in two limits of symmetric $G_1 = G_2$ (a, b) and highly asymmetric $G_1 = 0.1G_2$ (c, d) NSN device. Here we vary $G_{\rm th}$ which is responsible for the heat transmission between two adjacent NS junctions.
For pinched off or extra-long middle section of NSN device $G_{\rm th} \ll G_{1/2}$, two NS junctions are almost decoupled from each other and the transmitted signal in fig. S12 b, d is negligible. At the same time, the reflected noise is following the well-known $e^* = 2e \to e$ crossover for diffusive NS junctions [S1] that means a complete sub-gap reflection of the heat flux from S terminal. Increasing $G_{\rm th}$ we allow some heat transmission towards the right N terminal which results in the reduced sub-gap effective charge $e^* < 2e$ (slope) of the reflected noise. Particularly for the asymmetric device, effective charge approaches a single value $e^* \to e$ as $G_{\rm th},\ G_2 \gg G_1$ in fig. S12c. This is expected since S terminal in this case is effectively shorted with the right N terminal which serves as a heat sink for sub-gap quasiparticles.
The lost portion of the reflected heat flux is evident from the transmitted signal in fig. S12b, d which increases with increasing $G_{\rm th}$ at given bias voltage for both symmetric and asymmetric NSN junctions. Being the strong function of thermal conductance, the non-local noise is suitable for accurate determination of $G_{\rm th}$.

Noise vs. Resistive thermometry

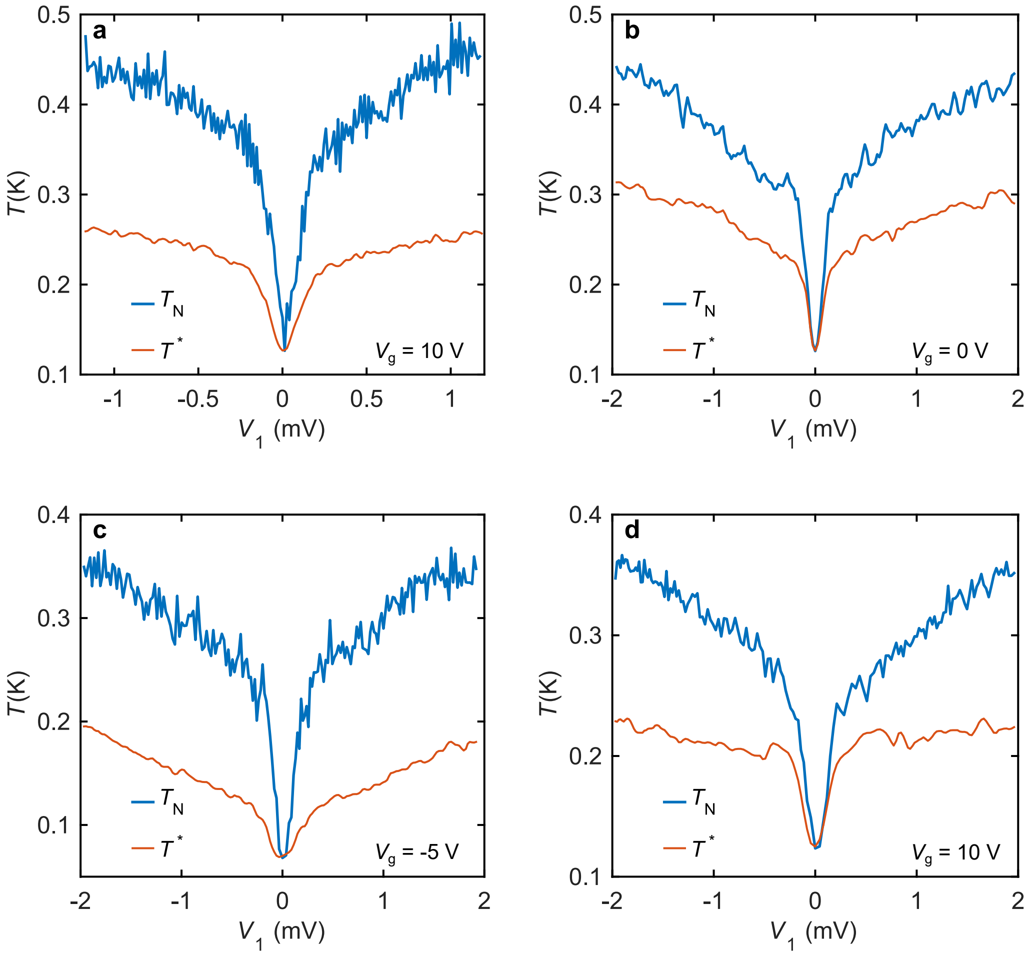

FIG. S13. **Comparison of the non-local noise thermometry and resistive thermometry.** (a) - (b) Measured noise and effective temperature of the floating NS junction (NSN - II device) at different back - gate voltages. Zero-bias thermometry underestimates noise one by the factor of ~ 2.

Is this section we highlight the difference between noise thermometry and resistive thermometry approaches.

To describe numerically the resistive thermometry, we consider NS junction as a two-terminal coherent quasi-1D conductor with n channels and energy-dependent diagonal transmission coefficients $Tr_i(\varepsilon)$ connecting two reservoirs with temperatures T_{L} and T_{R}. We now determine the linear response conductance G_1 in such a system, where a temperature bias across the conductor might be present.

If a small voltage bias dV is applied across the conductor, the linear-response current through the nanowire dI is [S3]:

$$dI = \frac{2e}{h}\sum_i^n \int Tr_i(\varepsilon)\left[f\left(\varepsilon - \frac{edV}{2}, T_{\mathrm{R}}\right) - f\left(\varepsilon + \frac{edV}{2}, T_{\mathrm{L}}\right)\right] d\varepsilon, \tag{S11}$$

where $f(\varepsilon, T)$ is the equilibrium Fermi-Dirac distribution at temperature T. We note, that the conductance temperature dependence in this model is fully enclosed in the energy dependence of $Tr_i(\varepsilon)$. Now we transform this equation by taking out the small bias from the distribution functions and separating the sum:

$$dI = \frac{2e}{h} \int \left[\sum_{i}^{n} Tr_i(\varepsilon) \right] \left[\frac{\partial f\left(\varepsilon, T_\mathrm{R}\right)}{\partial \varepsilon} \left(-\frac{edV}{2} \right) - \frac{\partial f\left(\varepsilon, T_\mathrm{L}\right)}{\partial \varepsilon} \left(\frac{edV}{2} \right) \right] d\varepsilon, \tag{S12}$$

which leads to the linear response conductance $G_{1,\mathrm{non-eq}}$ in form:

$$G_1 = \partial I / \partial V = -\frac{2e^2}{h} \frac{1}{2} \int \left[\sum_{i}^{n} Tr_i(\varepsilon) \right] \left[\frac{\partial f\left(\varepsilon, T_\mathrm{R}\right)}{\partial \varepsilon} + \frac{\partial f\left(\varepsilon, T_\mathrm{L}\right)}{\partial \varepsilon} \right] d\varepsilon = \frac{G_1(T_\mathrm{R}) + G_1(T_\mathrm{L})}{2}. \tag{S13}$$

where $G_1(T)$ is the ordinary equilibrium conductance which can be measured by varying the bath temperature. The (S13) clearly demonstrates, that the temperature, measured via resistive thermometry T^* in the main text obeys a relation:

$$G_1(T^*) = \frac{G_1(T_\mathrm{R}) + G_1(T_\mathrm{L})}{2}, \tag{S14}$$

We note, that this result is only valid for the case of equilibrium distribution functions at both nanowire terminals. For the triple-step distribution expected for S terminal in our experiment, the conductance depends on the exact form of $Tr_i(\varepsilon)$.

We now compare this result for T^* with the expected noise temperature T_N when one terminal has significantly higher temperature ($T_\mathrm{R} \gg T_\mathrm{L}$). For this case T_N can be expressed in form $T_\mathrm{N} = \alpha T_\mathrm{R}$, with coefficient α depending on the shape of distribution function at the terminal [S4]. For the double-step distribution $\alpha = 2/3$, while in the case of equilibrium distribution $\alpha = (1 + ln(2))/2 \approx 0.56$.

Neglecting the non-linearity of of $G_1(T)$, which is present in experiment (see supplemental fig. S9b), we obtain that the relation $T_\mathrm{N} \approx 4/3 T^*$.

In experiment, however, the discrepancy between T_N and T^* is more prominent with $T_\mathrm{N} - T_\mathrm{bath} \approx 2(T^* - T_\mathrm{bath})$ (see supplemental fig. S13). Apart from the discussed earlier effect of the non-equilibrium distribution on G_1, this inconsistency might be related to the dephasing being present in nanowire. Such dephasing may effectively break the nanowire into several coherent section, with possibly different signs of temperature dependence, leading to a further dampening of T^* compared to T_N.

Non-local voltage generated by temperature bias

In order to describe the symmetric component of the non-local I-Vs presented in Fig. 4d of the main text we consider a thermoelectric generation of voltage. In the Landauer-Büttiker formalism, the conversion of the temperature bias to electric current is a result of the energy dependence of the eigen-channel transparencies $Tr_i(\varepsilon)$. Thermoelectric current generated in a short-circuited conductor can be written as:

$$I_{\mathrm{TE}} = \frac{2e}{h}\sum_i^n \int Tr_i(\varepsilon)\left[f\left(\varepsilon, T_{\mathrm{R}}\right) - f\left(\varepsilon, T_{\mathrm{L}}\right)\right] d\varepsilon \approx \frac{2e}{h}\sum_i^n Tr_i^{'} \int \varepsilon\left[f\left(\varepsilon, T_{\mathrm{R}}\right) - f\left(\varepsilon, T_{\mathrm{L}}\right)\right] d\varepsilon, \tag{S15}$$

where the quasiparticle energy ε is measured with respect to the chemical potential that is the same for the right and left leads of the conductor. Note that in this equation we approximated the energy dependence $Tr_i(\varepsilon)$ with the lowest order non-vanishing term $Tr_i(\varepsilon) = Tr_i(0) + Tr_i^{'}\varepsilon$, where $Tr_i^{'} \equiv dTr_i(\varepsilon)/d\varepsilon|_{\varepsilon=0}$. It is straightforward to see that eq. (S15) results in a parabolic T-dependence of I_{TE}:

$$I_{\mathrm{TE}} \approx \frac{\pi^2 k_B^2}{6}\frac{2e}{h}\sum_i^n Tr_i^{'}\left[T_{\mathrm{R}}^2 - T_{\mathrm{L}}^2\right]. \tag{S16}$$

Measured thermoelectric voltage that builds up on a floating conductor is simply $V_{\mathrm{TE}} = -I_{\mathrm{TE}}G_0^{-1}$, where $G_0 = 2e^2/h\sum_i^n Tr_i(0)$ is the linear-response conductance. For a small temperature difference $\Delta T \equiv T_{\mathrm{R}} - T_{\mathrm{L}} \ll T$, the eq. (S16) can be written as:

$$V_{\mathrm{TE}} \approx (S/T)T\Delta T, \text{ where } S/T = -\frac{\pi^2 k_B^2}{3e}\sum_i^n Tr_i^{'}\left[\sum_i^n Tr_i\right]^{-1}. \tag{S17}$$

In other words, in this approximation the thermoelectric response is fully characterized by the T-independent Seebeck parameter S/T. With this notation we obtain the expression for arbitrary thermal biases on the conductor:

$$V_{\mathrm{TE}} \approx (S/T)\frac{T_{\mathrm{R}}^2 - T_{\mathrm{L}}^2}{2}, \tag{S18}$$

that is used to fit the data for V_2^{symm} in Fig. 4d of the main text.

Critical Temperature of Al contacts

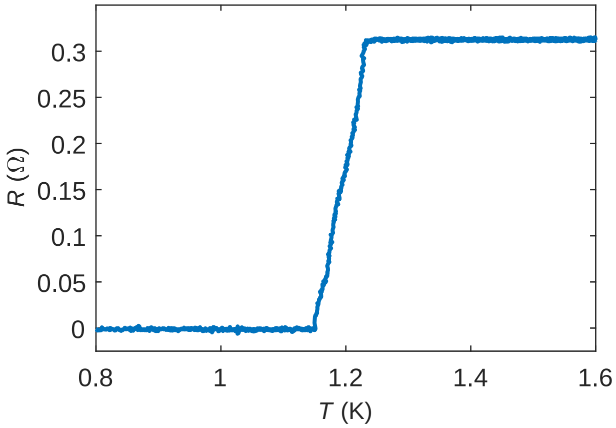

FIG. S14. **Superconducting critical temperature of the Al-film.** The resistance of a four-terminal Al strip, deposited via the same process, as the one used in the fabrication of the samples, featured in the main text.

The temperature dependence of superconducting Al, deposited via the same process as described in "Device Fabrication" was performed separately on the four-terminal Al strips, incorporated in the samples studied in [S5]. Here we present raw data (see Fig. S14), which leads to the estimate $T_c = 1.20 \pm 0.03\,\mathrm{K}$, based on the position of the middle of transition.

Device fabrication

InAs nanowires grown by molecular beam epitaxy on Si substrate [S6] are ultrasonicated in isopropyl alcohol. Nanowires are drop casted on Si/SiO2 (300 nm) substrates [S7] with preliminary defined alignment marks. For superconducting contacts conventional electron beam lithography (EBL) followed by e-beam deposition of Al (150 nm) is utilized. To obtain the ohmic contacts, in-situ Ar ion milling is performed before Al deposition in a chamber with a base pressure below 10^{-7} mbar. Normal metal contacts are fabricated in two different ways (different device batches): magnetron sputtering or e-beam deposition. For sputtering (NS and NSN - I devices) in-situ Ar plasma etching is followed by sputtering of Ti/Au (5 nm/200 nm). Normal metal contacts Ti/Au (5 nm/150 nm) in device NSN - II are deposited in the same way as superconducting ones.

[S1] K. E. Nagaev and M. Büttiker, Semiclassical theory of shot noise in disordered superconductor–normal-metal contacts, Phys. Rev. B **63**, 081301 (2001).
[S2] K. Nagaev, On the shot noise in dirty metal contacts, Physics Letters A **169**, 103 (1992).
[S3] Y. Blanter and M. Büttiker, Shot noise in mesoscopic conductors, Physics Reports **336**, 1 (2000).
[S4] E. S. Tikhonov, D. V. Shovkun, D. Ercolani, F. Rossella, M. Rocci, L. Sorba, S. Roddaro, and V. S. Khrapai, Local noise in a diffusive conductor, Scientific Reports **6**, 30621 EP (2016), article.
[S5] A. V. Bubis, A. O. Denisov, S. U. Piatrusha, I. E. Batov, V. S. Khrapai, J. Becker, J. Treu, D. Ruhstorfer, and G. Koblmüller, Proximity effect and interface transparency in al/InAs-nanowire/al diffusive junctions, Semiconductor Science and Technology **32**, 094007 (2017).
[S6] S. Hertenberger, D. Rudolph, M. Bichler, J. J. Finley, G. Abstreiter, and G. Koblmüller, Growth kinetics in position-controlled and catalyst-free InAs nanowire arrays on si(111) grown by selective area molecular beam epitaxy, Journal of Applied Physics **108**, 114316 (2010).
[S7] J. Becker, S. Morkötter, J. Treu, M. Sonner, M. Speckbacher, M. Döblinger, G. Abstreiter, J. J. Finley, and G. Koblmüller, Carrier trapping and activation at short-period wurtzite/zinc-blende stacking sequences in polytypic inas nanowires, Phys. Rev. B **97**, 115306 (2018).
